# Case Report: Identification of a novel hemizygous *CFAP47* variant in a primary ciliary dyskinesia patient with dual ciliary and flagellar defects

**DOI:** 10.3389/fmed.2025.1574684

**Published:** 2025-06-25

**Authors:** Miao He, Wangji Zhou, Yixuan Li, Qiaoling Chen, Yaping Liu, Xinlun Tian, Xue Zhang

**Affiliations:** ^1^McKusick-Zhang Center for Genetic Medicine, State Key Laboratory for Complex Severe and Rare Diseases, Institute of Basic Medical Sciences, Chinese Academy of Medical Sciences, School of Basic Medicine, Peking Union Medical College, Beijing, China; ^2^Department of Pulmonary and Critical Care Medicine, State Key Laboratory of Complex Severe and Rare Diseases, Peking Union Medical College Hospital, Chinese Academy of Medical Sciences, Peking Union Medical College, Beijing, China; ^3^State Key Laboratory for Complex Severe and Rare Diseases, State Key Sci-tech Infrastructure for Translational Medicine, Peking Union Medical College Hospital, Beijing, China

**Keywords:** CFAP47, variant pathogenicity, primary ciliary dyskinesia, cilia, genetic testing

## Abstract

**Background:**

Primary ciliary dyskinesia (PCD) is a genetically heterogeneous ciliopathy caused by structural and functional abnormalities of motile cilia. Although over 50 PCD-associated genes have been reported, the genetic spectrum remains incomplete. *CFAP47*, a gene linked to multiple morphological abnormalities of the flagella, has recently been implicated in PCD; however, further case studies are needed to strengthen this conclusion.

**Methods:**

We investigated a male patient with suspected PCD who exhibited “9 + 2” ultrastructural abnormalities in both bronchial cilia and sperm flagella. Whole exome sequencing was performed to screen for pathogenic variants. The candidate variant was analyzed through bioinformatics tools, and *CFAP47* expression levels were quantified via qPCR in both patient-derived sperm and an *in vitro* expression plasmid model.

**Results:**

Whole exome sequencing identified a hemizygous missense variant, *CFAP47* (NM_001304548.2): c.3599T > A (p.Phe1200Tyr) in the patient. The pathogenicity of this variant was assessed through multiple *in silico* tools, with divergent predictions. Experimental validation revealed significantly decreased CFAP47 mRNA levels in the patient’s sperm and the HEK293 cells transfected with mutant plasmid compared to controls, suggesting impaired transcript stability.

**Conclusion:**

Our study proposes a novel *CFAP47* variant as a likely contributor to PCD, given its impact on mRNA expression. These findings strengthen the association between *CFAP47* and PCD pathogenesis and expand the mutation spectrum of this emerging disease gene.

## 1 Introduction

Primary ciliary dyskinesia (PCD) is a rare genetic ciliopathy characterized by clinical and genetic heterogeneity, caused by structural and functional defects in motile cilia (1, 2). Clinical manifestations of PCD encompass chronic respiratory phenotypes (e.g., sinusitis, bronchiectasis, chronic wet cough), otolaryngological abnormalities (nasal polyps, otitis media with effusion), reproductive dysfunction (infertility/subfertility), and laterality defects (3). The diagnosis of PCD relies on a comprehensive assessment that includes clinical evaluation and various tests, including nasal nitric oxide (nNO) measurement, transmission electron microscopy (TEM), high-speed video analysis (HSVA), and genetic testing (4). As research progresses and more pathogenic genes and variants are identified, genetic testing is expected to play an increasingly critical role in the diagnostic process (4).

Motile cilia exhibit a conserved “9 + 2” ultrastructure, consisting of nine peripheral doublet microtubules (DMTs) and a central pair (CP) of singlet microtubules; each DMT is composed of an A-microtubule and a B-microtubule (5). Ciliary motility is driven by dynein arms extending from the A-microtubule to interact with adjacent B-microtubule. The outer dynein arms (ODAs) generate microtubule sliding forces, while the inner dynein arms (IDAs), through their functional coordination with the nexin-dynein regulatory complex (N-DRC) that bridges adjacent DMTs, modulate ciliary beating by regulation of dynein activity (6). The CP coordinates ciliary beat orientation across adjacent cilia to ensure metachronal synchrony, and radial spokes (RS) connect the central apparatus and IDAs, sending signals to the N-DRC to regulate dynein activity (7). Defects in genes associated with motile cilia formation, structure, and function may all lead to PCD. To date, over 50 genes associated with PCD have been identified. The majority of PCD-associated genes are inherited in an autosomal recessive pattern (e.g., *DNAH5* and *CFAP300*), while others exhibit autosomal dominant inheritance (*FOXJ1* and *TUBB4B*) or X-linked recessive inheritance (*RPGR*, *PIH1D3*, and *OFD1*) patterns (8, 9).

Genes within the cilia- and flagella-associated protein (CFAP) family are closely linked to the function of motile cilia. Pathogenic variants in *CFAP74*, *CFAP298*, and *CFAP300* have been definitively linked to PCD pathogenesis (10, 11). Several genes within the CFAP family, such as *CFAP43* and *CFAP44*, are thought to be associated with multiple morphological abnormalities of the flagella (MMAF) by affecting sperm flagella (12). Interestingly, despite causing similar diseases, the molecular functions and localizations of CFAP family members exhibit considerable diversity. Through cryo-electron microscopy and artificial intelligence-enabled structure prediction, the structures of axonemes have been analyzed, revealing that the CFAP61, CFAP91, and CFAP251 proteins are components of RS (13). CFAP57 and CFAP43/44 function as docking factors for RS and IDA, separately (13). CFAP20 is localized to the core inner junction protein, regulating the beating of motile cilia and the functions of non-motile cilia (14, 15). Many members of the CFAP family still have unclear functions and unknown subcellular localizations.

*Cilia- and flagella-associated protein 47* (*CFAP47*) gene has been identified as being associated with MMAF. Liu et al. (16) suggest that deleterious variants in the *CFAP47* gene are responsible for abnormal sperm tail morphology and infertility in affected patients, while male mice with frameshift variants in the *CFAP47* gene display comparable phenotypes (16). In addition to the MMAF phenotype, pathogenic *CFAP47* variants are also correlated with abnormalities in the sperm head and annulus (17). Ge et al. (18) propose that *CFAP47* variants are associated with both MMAF and PCD-like respiratory symptoms, including sinusitis and bronchiectasis, suggesting a potential role in ciliary dysfunction (18). The *CFAP47* gene (NM_001304548), located on the X chromosome, encompasses a coding region of 9,564 base pairs. Despite its potential significance, the Human Gene Mutation Database (HGMD) currently lists only twelve variants of this gene. The mutation spectrum of the *CFAP47* gene requires further exploration and expansion.

In this study, we investigated a patient with clinical features suggestive of PCD. Whole exome sequencing (WES) identified a hemizygous variant in *CFAP47*, and subsequent functional analyses revealed its association with reduced CFAP47 mRNA expression in both transfected HEK293 cells and the patient-derived sperm sample. These findings nominate this variant as a strong candidate for PCD pathogenesis, though additional studies are warranted to fully characterize its functional consequences.

## 2 Materials and methods

### 2.1 Subject

A patient presented to Peking Union Medical College Hospital with symptoms of sinusitis, bronchiectasis, and infertility. Following a thorough phenotypic assessment and laboratory tests, a clinical diagnosis of PCD was made. Peripheral blood, bronchial mucosa, and seminal fluid samples were collected for further analysis. Written informed consent was obtained from the participants for study participation. The ethics review board of the institution approved the study.

### 2.2 Whole exome sequencing

WES was performed by MyGenostics (Beijing, China). Genomic DNA was extracted from peripheral blood using the QIAamp DNA Blood Kit (Qiagen, Germany). A total of 1 μg DNA was fragmented to an average size of 180 bp using a Bioruptor sonicator (Diagenode, Belgium), and paired-end sequencing libraries were prepared with the DNA sample prep reagent set 1 (New England Biolabs, United States), including end repair, adapter ligation, and PCR enrichment. The amplified DNA was captured use GenCap exome capture kit (MyGenostics). The libraries were sequenced on an Illumina HiSeq X Ten platform with paired-end 150 bp reads, achieving an average sequencing depth of >100×. Raw sequencing data (FASTQ format) were processed by fastq-mcf to remove Illumina sequencing adapters and low-quality reads (<80 bp). Clean reads were aligned to the GRCh37/hg19 human reference genome using BWA, and duplicated reads were removed using Picard tools. Variant calling was performed using GATK HaplotypeCaller with the following filters applied via GATK VariantFiltration: (a) Mapping quality < 30; (b) Total mapping quality zero reads < 4; (c) Approximate read depth < 5; (d) QUAL < 50.0; (e) Phred-scaled *p*-value > 10.0 for Fisher’s exact test for strand bias. Filtered variants (VCF format) were annotated using ANNOVAR with multiple databases, such as 1000 Genomes, ESP6500, dbSNP, EXAC, In-house (MyGenostics), HGMD, and predicted using SIFT, PolyPhen-2, MutationTaster, and GERP++. Pathogenicity classification followed the ACMG/AMP 2015 guidelines.

### 2.3 Sanger sequencing and bioinformatic analysis

The candidate variant of patient was confirmed by Sanger sequencing with the primers listed in Supplementary Table 1. The potential impact of the variant on protein function and structure was assessed by AlphaFold3^1^ and DUET^2^, and evaluated according to ACMG/AMP guidelines.

### 2.4 Transmission electron microscopy

Semen samples were collected from both the patient and healthy control. Freshly collected specimens were first subjected to complete liquefaction at 37°C for 60 min to dissolve seminal coagulum, resulting in a homogeneous liquid phase. The liquefied semen was then centrifuged at 2000 rpm for 5 min to pellet spermatozoa, after which the seminal plasma supernatant was carefully aspirated. To remove residual seminal components, the pelleted spermatozoa underwent three sequential washes with 1 × phosphate-buffered saline (PBS, pH = 7.4), each consisting of gentle resuspension followed by re-centrifugation under identical conditions. A portion of the resulting pellet was fixed in 2.5% glutaraldehyde overnight for TEM analysis. The bronchial mucosa of the patient was obtained via bronchoscope and fixed using the same method. The bronchial mucosa and a portion of the sperm pellet were then post-fixed with 1% osmium tetroxide, dehydrated through a graded series of alcohol (30, 50, 70, 80, 95, and 100%) and acetone (100%), and infiltrated with a mixture of acetone and SPI-Pon 812 epoxy resin monomer. Following embedding in medium 812, the resin blocks were sectioned into 1.5 μm semi-thin slices using a semi-thin sectioning machine. These sections were stained with toluidine blue and examined under a light microscope. Subsequently, the resin blocks were further sectioned into ultra-thin slices of 60–80 nm using an ultra-thin sectioning machine, with the slices collected on a 150 mesh copper grid. The copper grids were stained with a saturated solution of uranyl acetate in 2% alcohol and a 2.6% lead citrate solution. Observations and imaging were performed using a transmission electron microscope (HITACHI HT7700; HITACHI HT7800) and the Hitachi TEM imaging system.

### 2.5 HE staining

A portion of the sperm pellet from section “2.4 Transmission electron microscopy” was resuspended in 1 × PBS, smeared onto adhesive slides, and fixed in 4% paraformaldehyde at room temperature for 30 min. The sperm smears were then stained with hematoxylin, followed by differentiation using a differentiation solution and bluing with a bluing solution. After dehydration with 95% ethanol, eosin staining was performed. Subsequent dehydration was conducted sequentially with anhydrous ethanol, n-butanol, and xylene. Finally, the slides were mounted with neutral gum. The sections were scanned using the Pannoramic MIDI, Pannoramic 250FLASH, and Pannoramic DESK systems from 3DHISTECH.

### 2.6 Construction and transfection of wild-type (WT) and mutant expression plasmids

The pcDNA3.1-*CFAP47*-WT-3 × Flag and pcDNA3.1-*CFAP47*-T3599A-3 × Flag expression vectors were constructed by Youbio (Changsha, China) using the pcDNA3.1 backbone, which contained the full-length coding sequence of *CFAP47* (RefSeq transcript NM_001304548.2). The mutant plasmid specifically introduced the c.3599T > A (p.Phe1200Tyr) variant through site-directed mutagenesis. HEK293 cells were maintained in MEM (Eagle’s Minimum Essential Medium) supplemented with 10% fetal bovine serum (FBS, Gibco) under standard culture conditions (37°C, 5% CO_2_). Transient transfection was performed at 80% confluence (approximately 5 × 10^5^ cells/well in 6-well plates) using the Lipofectamine 3000 Transfection Kit (Invitrogen), with 1 μg plasmid DNA per transfection. Cells were harvested 48 h post-transfection for downstream analyses.

### 2.7 RNA isolation and real-time quantitative PCR (qPCR)

A portion of the sperm pellet from section “2.4 Transmission electron microscopy”, along with HEK293 cells that were transfected for 48 h, were lysed using TRIzol reagent (Invitrogen) for total RNA extraction. cDNA was synthesized using the PrimeScript RT Master Mix (TaKaRa) according to the manufacturer’s protocol. qPCR was conducted with the Hieff qPCR SYBR Green Master Mix (Low Rox Plus) (YEASEN) employing a two-step amplification procedure: an initial denaturation at 95°C for 5 min, followed by 40 cycles of 10 s at 95°C and 30 s at 60°C. The sequences of the qPCR primers are provided in Supplementary Table 1. The mRNA expression level of *CFAP47* gene was normalized to that of *GAPDH*.

### 2.8 Statistics

All experiments were conducted at least three times. Results are presented as means ± Standard Error of the Mean (SEM). Statistical analysis was performed using GraphPad Prism version 8.0, and group differences were assessed using Student’s *t*-tests. A *P*-value of less than 0.05 was considered to indicate statistical significance.

## 3 Case description

### 3.1 Clinical manifestations

**FIGURE 1:**
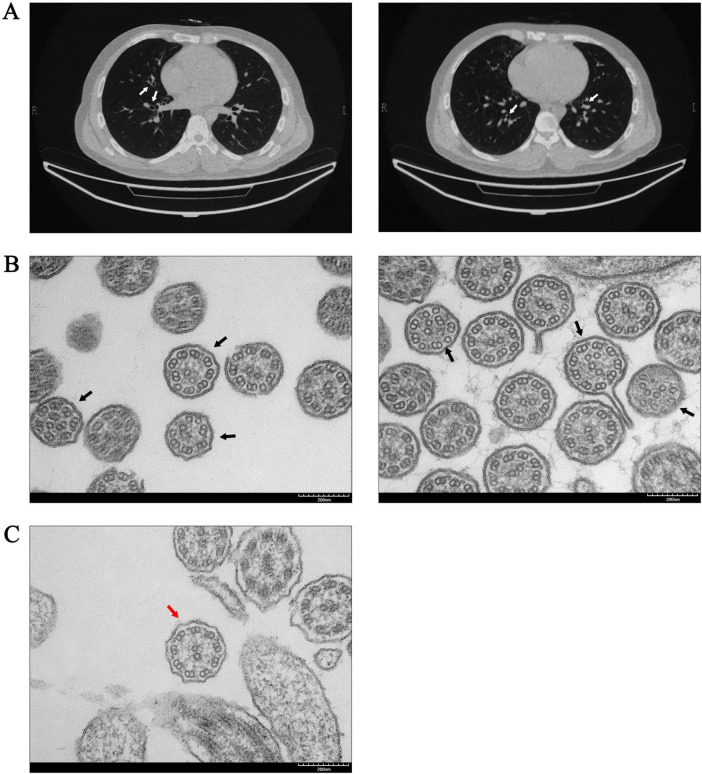
Abnormal manifestations in the respiratory system of the patient. **(A)** The chest computed tomography of the patient indicated bronchiectasis (white arrows). **(B)** TEM of the patient’s bronchial mucosa revealed abnormalities in the “9 + 2” microtubule structures. The central microtubules and peripheral doublet microtubules exhibited disordered arrangements, along with structural or numerical abnormalities (black arrows). **(C)** “9 + 2” ultrastructural arrangement of bronchial mucosa in a non-PCD control was normal (red arrow).

The patient was a 28-year-old man who visited Peking Union Medical College Hospital due to sinusitis, bronchiectasis, and infertility. He has suffered from sinusitis for nearly 20 years and has undergone three sinus surgeries; however, his nasal congestion and runny nose symptoms have not improved. Three years ago, a semen analysis revealed immotile sperm, and 2 years ago, a chest computed tomography scan identified mild bronchiectasis in the right middle lobe and both lower lobes (Figure 1A). A nNO test indicated a level of 7.2 ppb. Based on the clinical phenotypes, the suspected diagnosis for the patient was PCD, with no family history of the condition.

**FIGURE 2:**
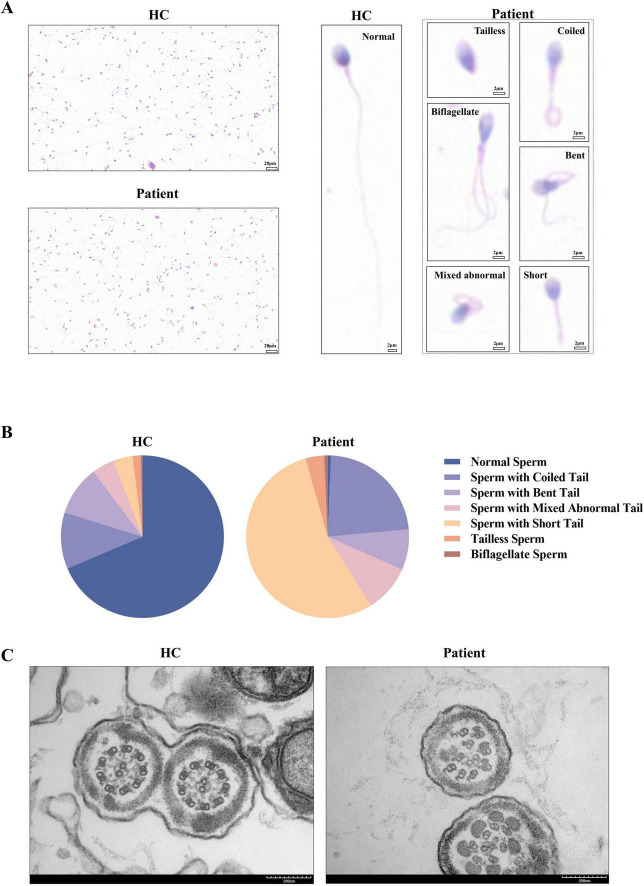
Abnormalities in the patient’s sperm. **(A,B)** Compared to the healthy control, the sperm of the patient exhibited coiled tails, bent tails, short tails, and so on in HE staining, with less than 1% of the sperm being normal after counting over 200 sperm. **(C)** The TEM analysis of sperm from the healthy control demonstrated the characteristic “9 + 2” microtubule structures. In contrast, the sperm flagella of the patient exhibited defects or disordered arrangements of the doublet microtubules, central microtubules, and peripheral dense fibers. HC, healthy control.

Sperm smears from both the patient and healthy control were stained using HE staining. Following the laboratory manual for human semen examination and processing, over 200 sperms were counted. The results indicated that the morphology of the sperm tails in the patient exhibited significant abnormalities, characterized by coiled tails, bent tails, short tails, and so on (Figure 2A). The patient presented with severe teratozoospermia (<1% morphologically normal spermatozoa), falling below the 4% diagnostic threshold defined in the World Health Organization’s Laboratory Manual for the Examination and Processing of Human Semen (5th edition) (Figure 2B). The TEM analysis revealed that, in contrast to the typical “9 + 2” microtubule arrangement observed in healthy control, all sperm flagella of the patient exhibited deficiencies or disordered arrangements of doublet microtubules, central microtubules, and peripheral dense fibers (twenty sperm flagella were observed) (Figure 2C). The TEM analysis of the bronchial mucosa demonstrated that the central microtubules in the patient were displaced or exhibited abnormal quantities. Additionally, the doublet microtubules displayed disordered arrangements, structural abnormalities, or variations in quantity, with some being either increased or decreased (Figure 1B). Approximately 50% of the cilia in the patient were abnormal (fifty cilia were observed). Figure 1C showed the ultrastructure of cilia from a non-PCD control. These findings provided additional support for the diagnosis of PCD.

### 3.2 Identification of a hemizygous CFAP47 missense variant

**FIGURE 3:**
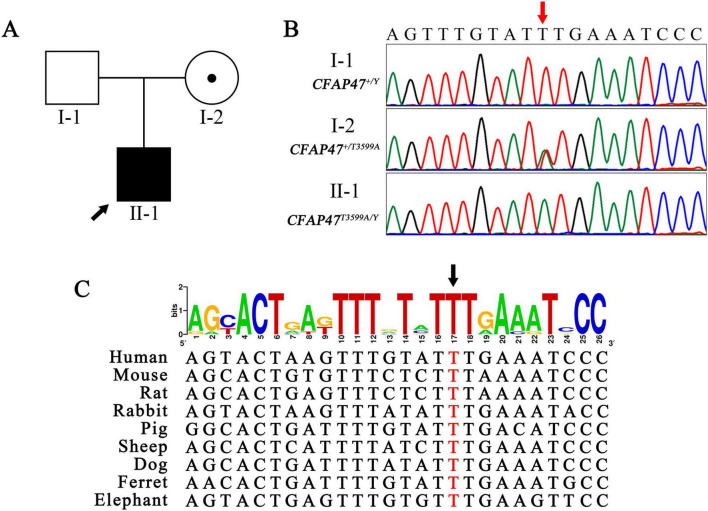
Maternal hemizygous variant conserved across species. **(A,B)** The pedigree and Sanger sequencing showed the patient carrying a hemizygous missense variant (red arrow) of the *CFAP47* gene inherited from his mother. Black filled square, patient; black arrow, proband. **(C)** The T3599 site (black arrow) was highly conserved among different species. The height of each letter in the figure is proportional to the occurrence frequency of the corresponding base or amino acid residue at that position, measured in bits.

WES analysis identified a hemizygous variant, *CFAP47* (NM_001304548.2): c.3599T > A (p.Phe1200Tyr) in the *CFAP47* gene in the patient, which was validated by Sanger sequencing (Figure 3B). No compound heterozygous or homozygous variants in other known PCD genes were found. This variant was inherited from his mother (Figure 3A). T3599, a conserved site among various species, was positioned within exon 23 of the *CFAP47* gene (Figure 3C). The occurrence of the c.3599T > A variant in the population is rare, with no recorded frequency in the gnomAD database^3^. Pathogenicity predictions for this variant were limited: AlphaMissense predicted it to be “Uncertain”, while DANN assessed it as “Deleterious”^4^. Other prediction tools, such as REVEL and SIFT, lacked available data for this specific variant. According to the ACMG/AMP guidelines, the c.3599T > A variant is classified as a variant of uncertain significance (VUS) (PM2).

### 3.3 Decreased CFAP47 expression in patient spermatozoa and transfected HEK293 cells

**FIGURE 4:**
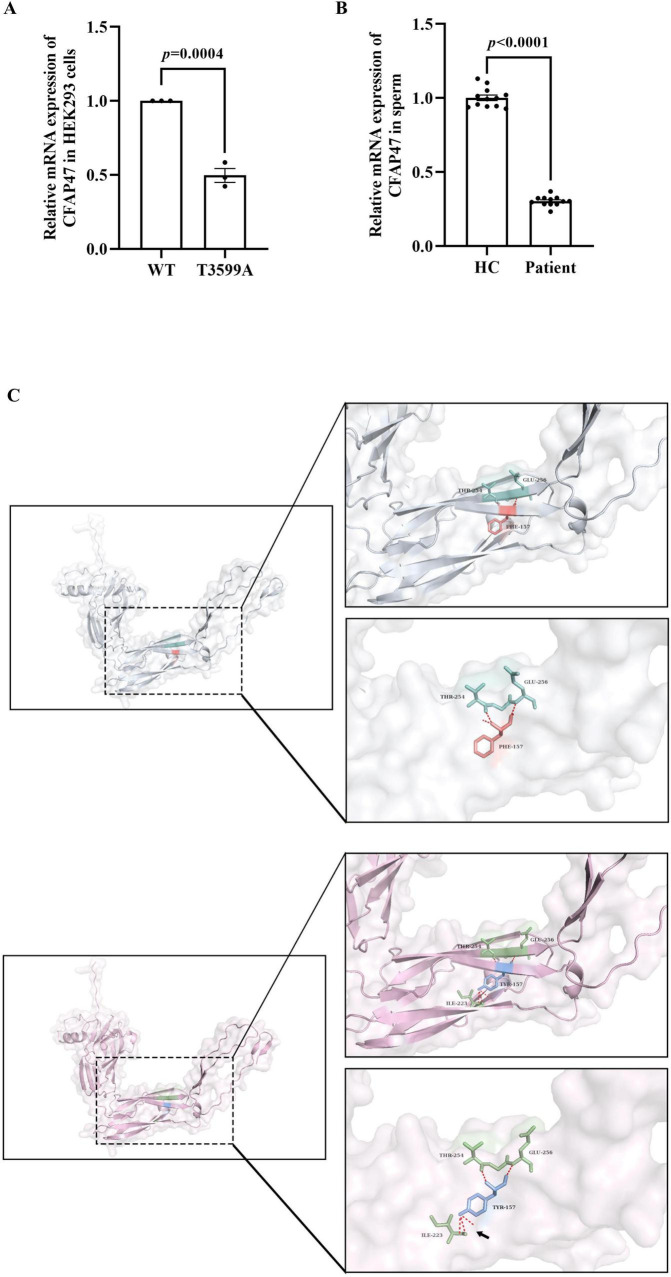
Effects of the variant on mRNA expression and potential impacts on protein structure. **(A)** In HEK293 cells transfected with the mutant plasmid, the expression level of CFAP47 mRNA was lower than that in cells transfected with the WT plasmid. Data represent the means±SEM, analyzed using the unpaired *t*-tests. Each black dot represents a biological repetition. **(B)** The expression level of CFAP47 mRNA in the patient’s sperm was lower than that of healthy control. Each black dot represents a technical repetition. HC, healthy control. **(C)** The structure of the WT-CFAP47 (in gray) from amino acids 1044–1356 was predicted using AlphaFold3. The F1200 residue (in orange) formed hydrogen bonds (in red) with the amino acids at positions 1297 and 1299 (in cyan). In contrast, when the amino acid was substituted with tyrosine in the mutant protein (in pink), a new hydrogen bond (black arrow) was established between Y1200 (in blue) and the amino acid at position 1266 (in green).

To assess the impact of the c.3599T > A variant on the *CFAP47* gene, qPCR was conducted in HEK293 cells transfected with expression plasmids, as well as in sperm samples from healthy control and patient. The findings revealed a reduced expression level of CFAP47 mRNA in the patient’s sperm compared to that in healthy control. Similarly, in HEK293 cells transfected with the mutant plasmid, the CFAP47 mRNA level was lower than that in cells transfected with the WT plasmid (Figures 4A, B).

### 3.4 Analysis of potential structural alterations in CFAP47 protein

The variant identified in the patient is p.Phe1200Tyr, which involves a substitution at the amino acid 1200, changing the hydrophobic non-polar amino acid phenylalanine to the hydrophilic polar amino acid tyrosine. Given limitations in protein expression assays, we employed AlphaFold3 to predict the structure of the CFAP47 protein (UniProt ID: Q6ZTR5-5)^5^ spanning amino acid residues 1044–1356, in order to assess the potential impact of the variant on protein structure. Compared to the WT protein, the mutant protein formed an additional hydrogen bond between amino acid 1200 and amino acid 1266, whereas the WT protein only formed two hydrogen bonds with amino acids 1297 and 1299 (Figure 4C). Subsequently, we utilized DUET to evaluate the effects of variant on protein stability, revealing that “Protein Stability Change Upon Mutation” (Supplementary Table 2).

## 4 Discussion

Primary ciliary dyskinesia is a disease characterized by high genetic heterogeneity. In diagnostic testing for clinical purposes, methods such as nNO measurement, TEM, and HSVA have strict requirements for specimen sampling, processing, and analysis, as well as the experience of the laboratory personnel. When the use of these methods is limited, the referential value of genetic testing will significantly increase (19). In a patient with a presumptive diagnosis of PCD exhibiting typical respiratory and reproductive system phenotypes, we identified a hemizygous missense variant in the *CFAP47* gene, c.3599T > A (p.Phe1200Tyr). This variant was associated with decreased CFAP47 mRNA expression in the patient’s sperm and in HEK293 cells transfected with mutant plasmid. To further validate its functional impact, experiments in ciliated cell models are critical. For example, introducing the variant into RPE-1 cells through CRISPR-Cas9 or stable transfection could assess evaluate its impact on ciliogenesis. Meanwhile, introducing it into 16HBE cells would facilitate the simultaneous evaluation of ciliary assembly and motility by air-liquid interface culture. Quantitative analysis of cilia length and beat dynamics in these models, combined with rescue experiments in patient-derived cells, would clarify whether the variant directly impairs CFAP47’s role in ciliary function. Although further functional validation is needed to definitively establish its pathogenicity, these findings enhance our understanding of the *CFAP47* mutation spectrum and strengthen its association with PCD pathogenesis.

Previous studies have demonstrated that *CFAP47* is highly expressed in the testes and is identified as being associated with MMAF (16, 17). Further research has confirmed *CFAP47* to be a potential gene responsible for PCD, and the expression is also observed in the lungs, trachea, and brain of mice (18). While the localization and function of *CFAP47* in sperm flagella has been well-documented in previous studies, its specific involvement in respiratory ciliary function remains to be elucidated. If *CFAP47* dysfunction is confirmed to be implicated in PCD, it becomes imperative to investigate the pathophysiological mechanisms underlying the phenotypic divergence whereby affected individuals manifest either MMAF or PCD clinical presentations. The patient in this study has a long-standing history of respiratory infections and bronchiectasis, with TEM revealing abnormalities in the “9 + 2” microtubule structure. Unfortunately, due to the severity of the patient’s infection, HSVA analysis was unable to capture ciliated cells. The decrease in nNo may be related to diffuse panbronchiolitis and respiratory tract infection (20, 21). Additionally, the ultrastructural changes in cilia observed via TEM may be influenced by respiratory infection and environmental factors (22). Further investigation is needed to understand the underlying causes of the respiratory phenotypes observed in our patient. Mori et al. (23) proposed that *CFAP47* may serve as a pathogenic gene for X-linked polycystic kidney disease (PKD), with the KO mice exhibiting vacuolation of tubular cells and tubular dilation (23). PKD is characterized by dysfunction of the primary cilia in renal epithelial cells (24). Notably, the *OFD1* gene, which is associated with X-linked PKD, can also lead to PCD when variants occur at the C-terminus (25, 26). This suggests that *CFAP47* may exert complex effects on various types of cilia across multiple organ systems.

The currently identified domain of the CFAP47 protein is the calponin-homology (CH) domain, spanning amino acids 1746–1869 (Supplementary Figure 1) (16). The variants reported in existing databases don’t reside within this domain, suggesting that the structural characteristics of the CFAP47 protein remain inadequately defined. Furthermore, the scattered distribution of variants across the gene implies that the pathogenicity associated with the *CFAP47* gene is likely attributable to loss of function. Consistent with this, functional analyses have confirmed that most pathogenic variants in the *CFAP47* gene lead to disease by reducing expression levels (16–18, 27). Interestingly, if the *CFAP47* gene is pathogenic due to loss of function, there are currently no records or reports of nonsense variants, indicating that the mutation spectrum and pathogenic mechanisms of *CFAP47* still need to be refined and explored. Our findings suggested that the c.3599T > A (p.Phe1200Tyr) variant may influence *CFAP47* gene expression, though further validation with additional healthy controls was warranted to establish definitive conclusions. The observed decrease in mRNA levels may be due to the instability of the CFAP47 transcript, potentially achieved by altering mRNA secondary structures or by generating new binding sites for RNA destabilizing factors. To validate these hypotheses, RNA stability assays, such as Actinomycin D chase experiments, could be employed to quantify the half-lives of mutant versus WT CFAP47 mRNA in transfected HEK293 cells. Meanwhile, computational tools could assist in identifying variant-induced instability signals. Elucidating these mechanisms will clarify the molecular basis of *CFAP47* dysregulation in this patient and establish a direct functional link between the variant and the pathogenesis of PCD. Additionally, AlphaFold3 structural predictions further suggested that this substitution introduces additional hydrogen-bonding interactions with position 1200, potentially destabilizing the CFAP47 protein—a plausible contributor to its pathogenicity.

Members of the CFAP family associated with PCD exhibit a range of diverse functions. CFAP46, CFAP54, CFAP74, and CFAP221 serve as structural proteins situated at the C1d projection of the central apparatus (11). The Chlamydomonas homolog of CFAP298, known as FBB18, is localized in the cytoplasm and is an integral component of the dynein preassembly factors interacting network, facilitating the proper assembly of ODAs and IDAs in cilia (28). Similarly, CFAP300 plays a critical role in the assembly of dynein arms (29). The precise function of the CFAP47 protein remains to be elucidated. CFAP47 possesses a CH domain, which is known to exhibit functional diversity through various protein interactions (30). CFAP47 may influence the morphological development of sperm by regulating the expression of CFAP65, CFAP69, and SEPTIN4 (17). Moreover, WDR87 interacts with CFAP47, forming a complex that is essential for the development of the sperm cell tail (27). Collectively, these findings suggest that the pathogenic mechanisms associated with CFAP47 protein dysfunction may arise from alterations in ciliary structure or disruptions in protein interactions.

In conclusion, we report a novel *CFAP47* variant in a PCD patient exhibiting male infertility, characteristic respiratory manifestations, and profound ultrastructural defects in both sperm flagella and respiratory cilia, notably the disruption of the conserved “9 + 2” microtubule arrangement. This variant is associated with reduced CFAP47 mRNA expression and potentially diminished protein stability. Our discovery broadens the mutation spectrum of *CFAP47*, advances insights into its essential role in maintaining ciliary ultrastructural and functional integrity, and establishes a critical foundation for evidence-based genetic counseling in the affected family.

## Data Availability

The raw data supporting the conclusions of this article will be made available by the authors, without undue reservation.

## Footnotes

1
https://alphafoldserver.com

2
https://biosig.lab.uq.edu.au/duet/

3
https://gnomad.broadinstitute.org/

4
https://franklin.genoox.com/clinical-db/home

5
https://www.uniprot.org/
