# Supplementary material for: Case Report: Identification of a novel hemizygous CFAP47 variant in a primary ciliary dyskinesia patient with dual ciliary and flagellar defects
Source: Front Med (Lausanne). 2025 Jun 25;12:1574684. doi: 10.3389/fmed.2025.1574684 (PMC12237681; doi:10.3389/fmed.2025.1574684)
Supplement: Supplementary file 1 [file Table_1.docx]

Supplementary Material

# Supplementary Tables

## Supplementary Table 1

**Supplementary Table 1. Primers used for PCR and qPCR in this study**

| Primer names | Primer Sequences (5'-3') | Accession number | Intron/exon location |
| --- | --- | --- | --- |
| *GAPDH*-qF | CACCATCTTCCAGGAGCGAG | NM_002046 | Exon 4&5 |
| *GAPDH*-qR | CAGAGATGATGACCCTTTTGGC | NM_002046 | Exon 6 |
| *CFAP47*-qF | TTGTTTCACCCCAAAGCTAATG | NM_001304548 | Exon 6&7 |
| *CFAP47*-qR | ATCCATCTTTACTTCCTACGGACTC | NM_001304548 | Exon 7 |
| *CFAP47*-T3599A-F | TGTTATTTTATTTTCTGGTTGGGAA | NM_001304548 | Intron 22 |
| *CFAP47*-T3599A-R | GAAGAGCATAAACAATTGGGGAG | NM_001304548 | Intron 23 |

## Supplementary Table 2

**Supplementary Table 2. The impact of variant (p.F1200Y) on CFAP47 protein stability**

| Prediction Method | ΔΔG (kcal/mol) | Predicted Result |
| --- | --- | --- |
| mCSM | -0.493 | Destabilizing |
| SDM | -0.84 | Destabilizing |
| DUET | -0.386 | Destabilizing |

SDM: site‐directed mutator; mCSM: mutation cutoff scanning matrix; ΔΔG represents the change in free energy associated with a protein variant and is widely utilized to predict the effects of these variants on protein stability(Pandurangan and Blundell, 2020).

## Supplementary Table 3

**Supplementary Table 3. Pathogenic/Likely Pathogenic variants**

| Gene | Transcript | AA Change | Nucleotide | Zygosity | Confidence | Genoox Classification |
| --- | --- | --- | --- | --- | --- | --- |
| *PSEN2* | NM_000447.3 | p.Met298Lys | c.893T>A | het | High | Likely Pathogenic |
| *PSEN1* | NM_000021.4 | p.Thr281Met | c.842C>T | het | High | Likely Pathogenic |
| *SCNN1A* | NM_001038.6 | p.Arg600Serfs*125 | c.1797_1813del | het | High | Likely Pathogenic |
| *UGT1A3* | NM_019093.4 | p.Arg258* | c.772C>T | het | High | Likely Pathogenic |
| *B4GALNT3* | NM_173593.4 | p.Arg264* | c.790C>T | het | High | Likely Pathogenic |
| *CDC27* | NM_001256.6 | p.Ala593Thr | c.1777G>A | hom | Low | Likely Pathogenic |
| *HLA-DRB1* | NM_002124.4 | | c.370+1G>A | het | Low | Likely Pathogenic |
| *POMT1* | NM_001374689.1 | p.Arg267* | c.799C>T | het | High | Likely Pathogenic |
| *CCDC40* | NM_017950.4 | p.Arg321* | c.961C>T | het | High | Pathogenic |
| *PYGL* | NM_002863.5 | | c.772+1G>A | het | High | Pathogenic |
| *TPI1* | NM_000365.6 | p.Gly123* | c.367G>T | het | High | Likely Pathogenic |
| *ASPA* | NM_000049.4 | p.Cys152Ser | c.455G>C | het | High | Likely Pathogenic |
| *PIDD1* | NM_145886.4 | p.Arg501* | c.1501C>T | het | High | Likely Pathogenic |
| *HYDIN* | NM_001270974.2 | p.Gln3905Argfs*5 | c.11712del | het | Low | Pathogenic |

# Supplementary Figures

## Supplementary Figure 1


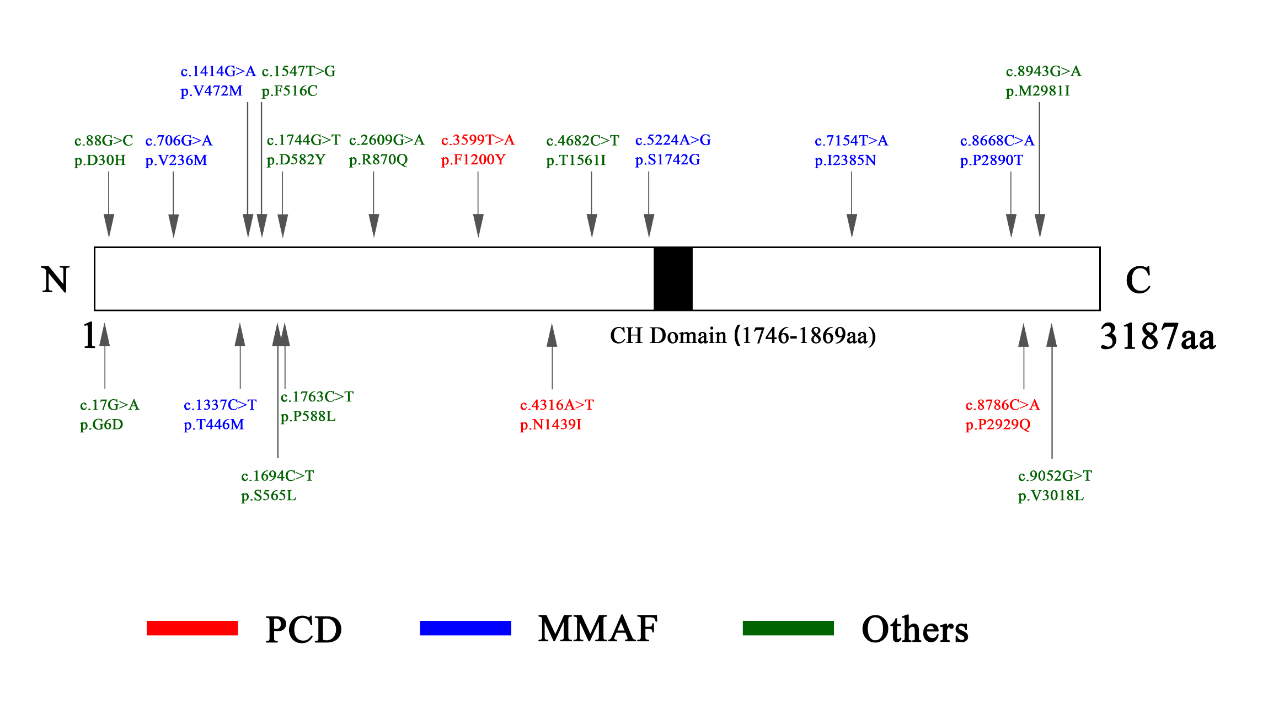


**Supplementary Figure 1.** Mutation spectrum of the *CFAP74* gene. The variants are distributed throughout the *CFAP47* gene, encompassing those associated with PCD (Ge et al., 2024), MMAF (Liu et al., 2021; Liao et al., 2022; Liu et al., 2023), and other diseases (Al-Mubarak et al., 2017; Kaplanis et al., 2020; Buonaiuto et al., 2022; Mori et al., 2024; Zhou et al., 2024). CH Domain, calponin-homology domain.

# Supplementary References

Al-Mubarak, B., Abouelhoda, M., Omar, A., AlDhalaan, H., Aldosari, M., Nester, M., et al. (2017). Whole exome sequencing reveals inherited and de novo variants in autism spectrum disorder: a trio study from Saudi families. *Sci Rep* 7(1)**,** 5679. doi: 10.1038/s41598-017-06033-1.

Buonaiuto, S., Biase, I.D., Aleotti, V., Ravaei, A., Marino, A., Damaggio, G., et al. (2022). Prioritization of putatively detrimental variants in euploid miscarriages. *Sci Rep* 12(1)**,** 1997. doi: 10.1038/s41598-022-05737-3.

Ge, H., Zhou, W., He, M., Zheng, H., Zhao, X., Zhang, T., et al. (2024). Mutations in CFAP47, a previously reported MMAF causative gene, also contribute to the respiratory defects in patients with PCD. *Mol Genet Genomic Med* 12(1)**,** e2278. doi: 10.1002/mgg3.2278.

Kaplanis, J., Samocha, K.E., Wiel, L., Zhang, Z., Arvai, K.J., Eberhardt, R.Y., et al. (2020). Evidence for 28 genetic disorders discovered by combining healthcare and research data. *Nature* 586(7831)**,** 757-762. doi: 10.1038/s41586-020-2832-5.

Liao, H.Q., Guo, Z.Y., Huang, L.H., Liu, G., Lu, J.F., Zhang, Y.F., et al. (2022). WDR87 interacts with CFAP47 protein in the middle piece of spermatozoa flagella to participate in sperm tail assembly. *Mol Hum Reprod* 29(1). doi: 10.1093/molehr/gaac042.

Liu, C., Tu, C., Wang, L., Wu, H., Houston, B.J., Mastrorosa, F.K., et al. (2021). Deleterious variants in X-linked CFAP47 induce asthenoteratozoospermia and primary male infertility. *Am J Hum Genet* 108(2)**,** 309-323. doi: 10.1016/j.ajhg.2021.01.002.

Liu, M., Dai, S., Zhang, J., Yang, Y., Shen, Y., Liu, H., et al. (2023). A novel mutation in CFAP47 causes male infertility due to multiple morphological abnormalities of the sperm flagella. *Front Endocrinol (Lausanne)* 14**,** 1155639. doi: 10.3389/fendo.2023.1155639.

Mori, T., Fujimaru, T., Liu, C., Patterson, K., Yamamoto, K., Suzuki, T., et al. (2024). CFAP47 is Implicated in X-Linked Polycystic Kidney Disease. *Kidney Int Rep* 9(12)**,** 3580-3591. doi: 10.1016/j.ekir.2024.09.013.

Pandurangan, A.P., and Blundell, T.L. (2020). Prediction of impacts of mutations on protein structure and interactions: SDM, a statistical approach, and mCSM, using machine learning. *Protein Sci* 29(1)**,** 247-257. doi: 10.1002/pro.3774.

Zhou, H., Yin, Z., Ni, B., Lin, J., Luo, S., and Xie, W. (2024). Whole exome sequencing analysis of 167 men with primary infertility. *BMC Med Genomics* 17(1)**,** 230. doi: 10.1186/s12920-024-02005-3.
